# The *Drosophila Over Compensating Males* Gene Genetically Inhibits Dosage Compensation in Males

**DOI:** 10.1371/journal.pone.0060450

**Published:** 2013-04-02

**Authors:** Chiat Koo Lim, Richard L. Kelley

**Affiliations:** 1 Program in Developmental Biology, Baylor College of Medicine, Houston, Texas, United States of America; 2 Department of Molecular and Human Genetics, Baylor College of Medicine, Houston, Texas, United States of America; CNRS UMR7275, France

## Abstract

Male *Drosophila* are monosomic for the X chromosome, but survive due to dosage compensation. They use the Male Specific Lethal (MSL) complex composed of noncoding *roX* RNA and histone modifying enzymes to hypertranscribe most genes along the X ∼1.6–1.8 fold relative to each female allele. It is not known how the MSL complex achieves this precise adjustment to a large and diverse set of target genes. We carried out a genetic screen searching for novel factors that regulate dosage compensation in flies. This strategy generated thirty alleles in a previously uncharacterized gene, *over compensating males* (*ocm*) that antagonizes some aspect of MSL activity. The mutations were initially recovered because they derepressed an MSL-dependent eye color reporter. Null *ocm* mutations are lethal to both sexes early in development revealing an essential function. Combinations of hypomorphic *ocm* alleles display a male specific lethality similar to mutations in the classic *msl* genes, but *ocm* males die due to excessive, rather than lack of dosage compensation. Males that die due to very low MSL activity can be partially rescued by *ocm* mutations. Likewise, males that would die from *ocm* mutations can be rescued by reducing the dose of various *msl* and *roX* genes. *ocm* encodes a large nuclear protein that shares a novel cysteine rich motif with known transcription factors.

## Introduction

*Drosophila* utilize a genetic mechanism to determine sex. Embryos with two X chromosomes develop into females while those with only one adopt a male phenotype [1]. Males escape the potentially lethal imbalance of X and autosomally encoded gene products by the process of dosage compensation. Males hypertranscribe most genes along the single X to match the output of females with two X chromosomes [2]–[4]. This is accomplished by the action of the massive MSL (male-specific lethal) complex that binds the male X at hundreds of sites, but not the male autosomes or any chromosome in females [5]. One mechanistic model postulates that the MSL complex acts at the elongation step of transcription of X linked genes [6], [7] although others favor action at recruiting RNA polymerase II to promoters [8].

The dosage compensation complex in *Drosophila* seems to have evolved from a general chromatin remodeling machine that in other species is responsible for most histone H4K16 acetylation [9], [10]. Histone acetylation is carried out by the MOF (males absent on first) subunit [11]. The MSL1 protein serves as a central assembly scaffold whose PEHE domain near the C-terminus recruits both MOF and the chromodomain protein MSL3 [12]–[15]. The N terminal coiled coil of MSL1 heterodimerizes with MSL2. The MSL3 protein seems to aid local cis spreading of the complex [16], [17], but the particular molecule recognized by its chromo domain is controversial [18]. The RING finger of MSL2 is an ubiquitin E3 ligase responsible for ubiquitylation of histone H2B at K34 [19]. It can also ubiquitinate itself for degradation to maintain proper stoichiometery between the MSL proteins [20]. A second cys-rich region of MSL2 has general affinity for DNA [21]. This may explain why MSL2 lacking its *roX* RNA partner binds all chromosomes indiscriminately [22]. Genes encoding homologs of these four proteins are found in other insects and vertebrates [23].

At least three major innovations were necessary to recruit the MSL proteins to the new function of hypertranscribing the male X in *Drosophila*. A new targeting mechanism was needed to limit the MSL complex to the X. This involved the large noncoding *roX* RNAs (RNA on X chromosome) and the MLE (maleless) helicase thought to play some role in folding *roX* RNA or assembling MSL proteins onto *roX* RNA [24]. MLE, the homolog of vertebrate RNA helicase A, is distinctive among helicases in having two dsRNA binding motifs at it N-terminus [25]. Next, the complex needed to be limited to males. This was accomplished by bringing translation of *msl2* mRNA under the negative control of the female-specific master regulator of sex determination, SXL (Sex Lethal) [26]. MSL2 expression was further limited to replication phase so that MSL complex could be made in a burst as the X chromatin substrate doubled [22]. Finally, the individual MSL proteins acquired adaptations needed for dosage compensation such as the large N-terminus of MOF and the extreme C terminus of MSL1, both found only within the genus *Drosophila* [14], [27]. A remarkable feature of this evolutionary history is that modern *Drosophila* females survive without the ancestral functions of the MSL proteins. The same is likely true for male autosomal genes. Each of the *msl* genes is single copy and as the name suggests, null mutations appear to have no impact on females. The exception is *mof* where null mutant females are viable, but recovered at only half the expected numbers [27]. MOF is also found in a second complex that may explain how the *msl* genes were freed to shift functions [28].

We are interested in finding any additional factors that may be needed to help the MSL proteins carry out their new dosage compensation function. Genetic screens looking for the male-specific lethal phenotype have probably reached saturation. Biochemical purification of the MSL complex requires stable protein interactions that survive extraction and has had limited success in finding new partners [29]. Recent cell culture RNAi screens searching for regulators of *roX2* have identified many promising new candidates in the dosage compensation pathway including a Zn finger protein, *CG1832*, that may provide the sequence specificity for targeting MSL complex to chromatin entry sites [30]. We developed a new genetic strategy in adult flies that relies on an eye color reporter sensitive to small changes in dosage compensation. We found that loss of function mutations in the newly identified *ocm* (*over compensating males*) gene cause elevated dosage compensation of the eye color reporter. Certain combinations of hypomorphic missense alleles display a male-specific lethality as seen with the classic *msl* genes. Our analysis demonstrates that *ocm* is a negative regulator of the MSL complex limiting dosage compensation to two-fold. Reduction of OCM selectively kills males due to excessive dosage compensation. Recovering a wide allelic series of *ocm* mutations allowed us to identify roles in oogenesis, spermatogenesis, and early development in addition to its role regulating the MSL complex.

## Results

### Isolating *Over Compensating Male* mutations

**Figure 1:**
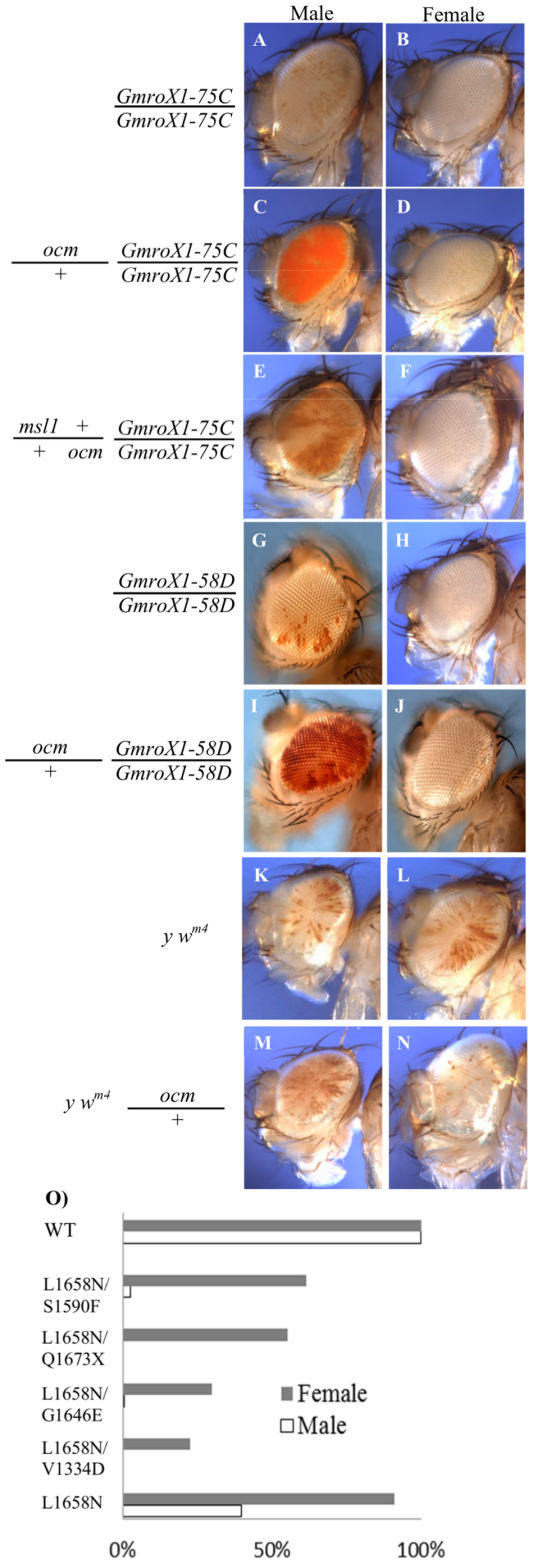
Mutations in *ocm* increase MSL activity around mosaic *roX1* transgenes. A and B. The *GMroX1-75C* reporter shows mosaic eye pigmentation in males, but females have pure white eyes. C and D. Reducing *ocm* activity increases eye pigmentation in males (more MSL activity) but has no effect on females who lack dosage compensation. E and F. The increased eye pigmentation seen in *ocm* males requires full MSL1 activity. G-J. A different *roX1* mosiac reporter displays the same male-specific *ocm* phenotype. K-N. Position effect variegation as measured by *In(1) w* ^m4^ is not affected by *ocm* mutations. The exact phenotypes of the flies are in the methods section. O. Several hypomorphic *ocm* allelic combinations produce abundant females, but few or no males. X axis is viability. Allelic designations indicate codons affected. See Table S2 for details.

Transgenes marked with the *miniwhite* eye color gene occasionally land in regions of repressive chromatin so that little or no eye pigment is made. If the transgene carries *roX1*, one of the genes producing the large noncoding RNA component of the MSL complex, the MSL complex is targeted to the transgene and acetylates surrounding chromatin so that the *miniwhite* gene is expressed [31]. Because dosage compensation is limited to males, only males have pigmented eyes, often in a mosaic pattern. Female eye color remains fully repressed. This was used as the starting point of a genetic screen looking for dominant mutations that altered the degree of male eye pigmentation. We previously described isolating unusual alleles of *msl1* and *mle* in this screen [14]. Here we characterize a new gene, the *166* complementation group, initially represented by three alleles recovered in a small pilot screen using the *GMroX1-75* mosaic eye reporter (Fig. 1A–D). Because of the male-specific phenotypes characterized below, we named this mutation *over compensating males (ocm)*. We repeated a modified version of the screen on a larger scale to recover a total of 30 mutations in *ocm* (Table S1A). Nineteen alleles were isolated using [*w* ^+^ *GMroX1-75C*] and the other eleven using a different mosaic reporter, [*w* ^+^ *GMroX1-58D*] (Fig. 1G–J). The reason for using different reporters was to increase the chances of isolating mutations affecting dosage compensation rather than chromatin silencing factors specific to a particular insertion site. *GMroX1-75C* is located within a yoyo repetitive element on the third chromosome, and *GMroX1-58D* is located in the 5′ end of the *dve* gene on the second chromosome. They are likely repressed by different modes of chromatin silencing.

### *ocm* Allelic Series Reveals Male-specific Lethality

Allelism of the newly isolated modifiers could be established by using a second phenotype, recessive lethality to both sexes. Although the eye color phenotypes were similar for all alleles, the mutations could be placed in a phenotypic series based on recovery of adult progeny with certain hypomorphic allelic combinations (Table S2). The increased eye pigmentation suggests elevated levels of dosage compensation around the reporter. An alternative interpretation is that the mutations instead act to generally relieve repressive chromatin allowing increased *miniwhite* expression. The fact that *ocm* mutations do not relieve repression in females argues against such a general function, and instead suggests that *ocm* somehow acts through the MSL dosage compensation system (Fig. 1D and J). To more directly examine this point, we crossed *ocm* to the *In(1)w^m4^* mutation that has been used extensively to assay for position effect variegation [32]. Mutations in *ocm* had no dominant effect on the speckled pigmentation in males or females (Fig. 1K–N) arguing against the idea that *ocm* somehow affects repressive chromatin packaging. The most striking finding was that several allelic combinations produced abundant females, but no males similar to the know *msl* components of the dosage compensation pathway (Fig. 1O, Table S2). Unlike *msl* mutants, *ocm* females were invariably sterile. The weakest allelic combinations produced some sons (Table S1), but they too were sterile.

All the *ocm* alleles recovered were loss of function (below). Changing the mosaic pattern to solid red suggested elevated dosage compensation locally around the *roX1* autosomal reporter when OCM levels were reduced (Fig. 1C and I). However, survival of these *ocm*/+ heterozygotes argued that dosage compensation of the X was within tolerable limits. If *ocm* produced solid red eyes by acting upon the MSL pathway, male eye color should be sensitive to the level of MSL complex. This suspicion was confirmed by making double mutants heterozygous for both *ocm* and *msl1*. The solid red eye pigmentation of *ocm*/+ males returned to a mosaic pattern when MSL1 levels fell (Fig. 1E and F). We conclude that *ocm* normally antagonizes some aspect of MSL dosage compensation because *ocm* mutations derepress the *miniwhite-roX1* reporter in males but not females, and this requires wild type levels of MSL1 protein.

### *ocm* Mutations Lead to Excess Dosage Compensation of the Male X

**Figure 2:**
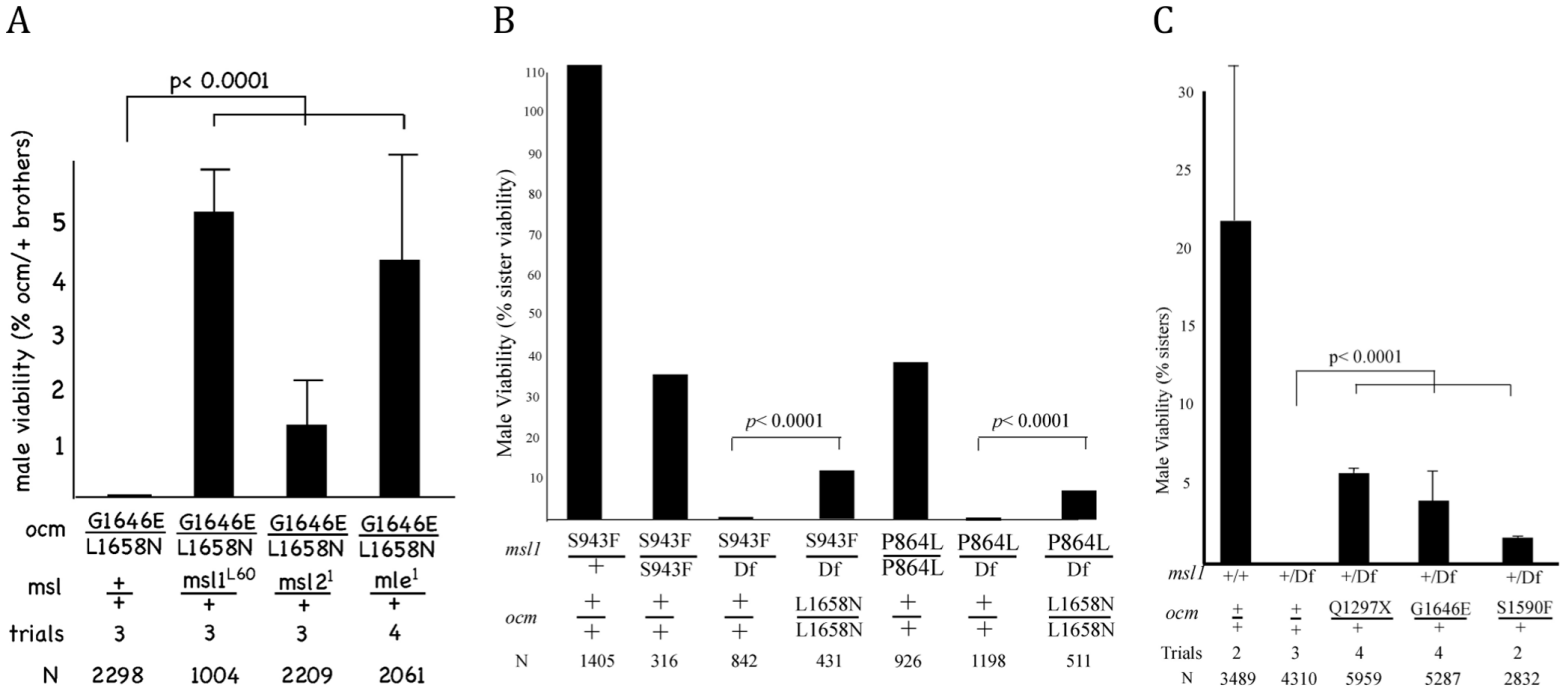
*ocm* antagonizes MSL activity. A. The male-specific lethal phenotype of hypomorphic *ocm* alleles are rescued by reducing levels of MSL subunits. Mutant male viability relative to brothers carrying *CyO* balancer. B. Two different hypomorphic alleles of *msl1* are viable when homozygous but dead when hemizygous [14]. Both alleles are partially rescued by reducing *ocm* activity. Viability of males is shown relative to sisters of the same genotype. C. *roX1 roX2/Y; [w^+^ GMroX1-75C]* females were mated to males donating either the *msl1* ^L60^ null allele (Df), or males carrying both *msl1* ^L60^ and the indicated *ocm* allele. Male viability relative to sisters with same autosomal genotype. Trials indicate independent crosses examined. N indicates number of relevant control siblings used to calculate viability. p values calculated by Fisher exact test.

We next asked if ordinary dosage compensation along the male X was also affected. The strategy used to initially recover *ocm* mutations required that *ocm*/+ males survive, so if dosage compensation of the X is affected, any change must be modest. Although null alleles of *ocm* were lethal to both sexes when homozygous, certain hypomorphic allelic combinations produced abundant females but no males (Fig. 1O and Table S2) that might indicate a problem with dosage compensation of the X chromosome. Male lethality caused by null mutations in the classic *msl* genes results from failure to hypertranscribe the male X [2]–[4], but see [33] for a different interpretation. If hypomorphic *ocm* males were instead dying from excessive transcription of the X, then reducing MSL levels should restore male viability. Figure 2A shows that nearly all *ocm* ^L1658N/G1643E^ males die, but their viability is partially restored in *msl1*/+, *msl2*/+, or *mle*/+ heterozygotes. This is consistent with the idea that low *ocm* levels results in toxic excess hyperactivation of the male X that can be partially corrected by reducing individual MSL subunits. In the course of these experiments we also produced females that were homozygous for each of the *msl* mutations as well as *ocm* ^L1658N^. These doubly mutant females failed to produce eggs demonstrating that ectopic dosage compensation could not be responsible for *ocm*’s toxic effect on female viability and oogenesis.

We also examined the converse situation of males dying due to low dosage compensation. Males homozygous for either the hypomorphic *msl1* ^P864L^ or *msl1* ^S943F^ allele are viable. However, each is male lethal in combination with a null *msl1* allele due to inadequate dosage compensation [14]. Figure 2B shows that reducing *ocm* activity partially rescues these males dying from insufficient MSL activity. A similar result was found for reduced *roX1* RNA, normally produced from an X-linked gene. When the endogenous *roX1* and *roX2* genes are deleted, males die because the MSL proteins are no longer targeted to the X chromosome [24]. Male viability can also be restored by an autosomal *roX1* or *roX2* transgene. However, autosomal supplied *roX1* RNA may not always be sufficient for normal levels of dosage compensation. For instance, *roX1 roX2*/Y; [*GMroX1-75*C]/+ males are recovered in much lower numbers compared to their sisters (Fig. 2C). Removing one copy of *msl1* further reduced MSL complex activity resulting in complete male lethality. However, three different alleles of *ocm* each help males overcome this deficit of MSL complex allowing some to reach adulthood (Fig. 2C). We proposed that one function of OCM is to limit the activity of MSL complex so that X-linked genes are hypertranscribed at the necessary two-fold level relative to females.

### *ocm* Mutations do not Alter MSL Distribution Along the Male X

Males carrying *ocm* ^L1658N/G1643E^ alleles reach third instar larvae, but die before adulthood. We examined their polytene chromosomes for changes in gross morphology that might result from excessive transcription of the X chromosome. The MSL staining pattern was not disrupted and no consistent abnormality was seen in the polytene banding pattern (Fig. S1A). Females mutant for *ocm* successfully repress MSL expression, excluding the idea that female toxicity is due to ectopic MSL expression (Fig. S1B). We also examined whether *ocm* mutations affected the ability of the MSL complex to spread in *cis* from autosomal *roX1* transgenes. Local MSL spreading is highly sensitive to the ratio of MSL subunits to nascent *roX* transcripts and may reflect some aspect of assembly of MSL complex [34], [35]. The MSL complex was limited to a single band over *roX1* transgenes (no spreading) when the X chromosome carried wild type alleles of *roX1* and *roX2* regardless of whether *ocm* was wild type or heterozygous (Fig. S1C–D). Similarly, massive local spreading of MSL complex occurred around *roX1* transgene when the endogenous *roX1* and *roX2* loci were deleted regardless of *ocm* status (Fig. S1E–F). Finding that MSL staining of the X is normal and *cis* spreading is not altered in *ocm* males suggests that production of MSL subunits and *roX* transcripts are not grossly affected by reduced OCM levels.

### *Over Compensating Males* Corresponds to *CG3363*

**Figure 3:**
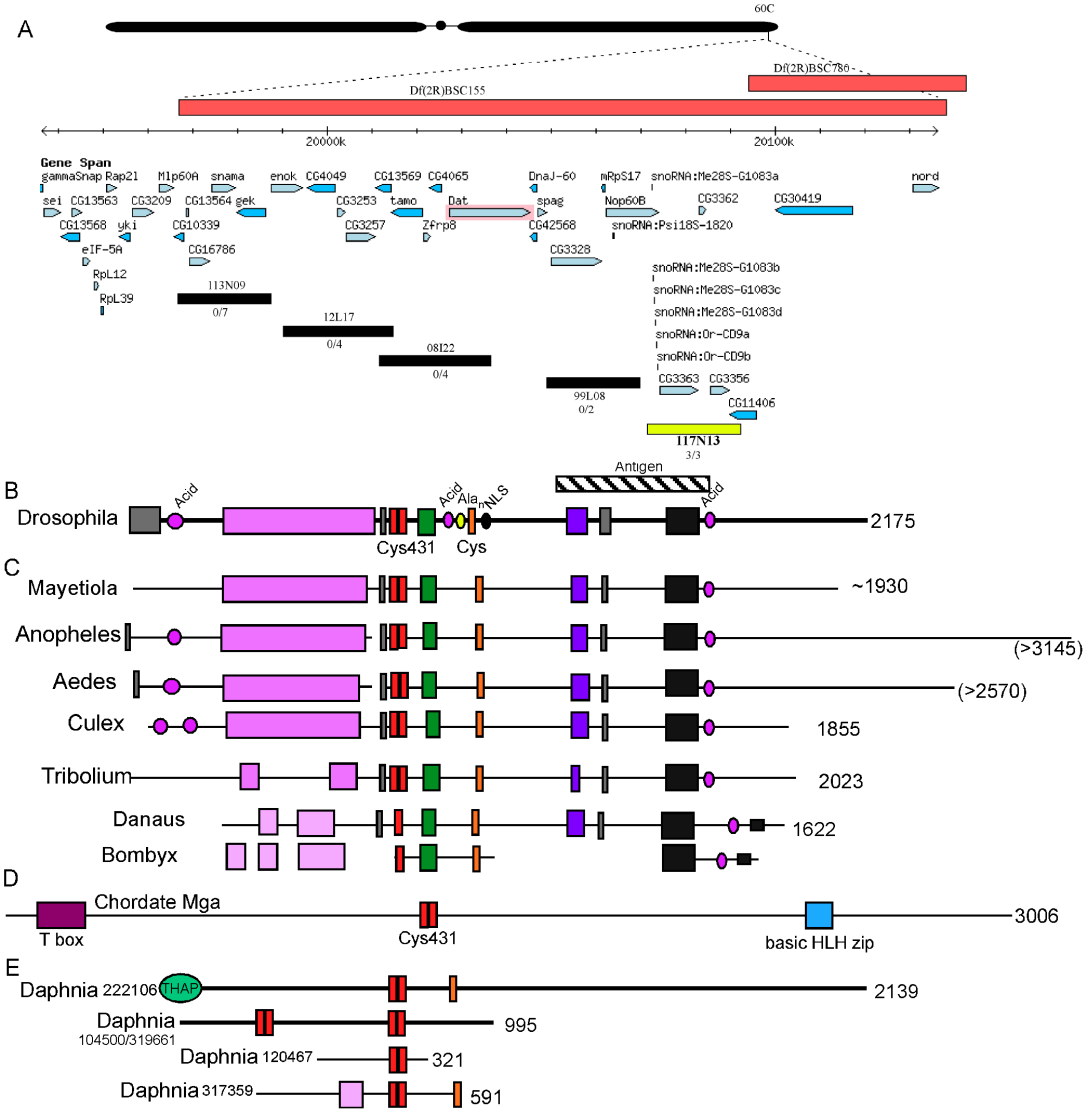
Identification of *CG3363* as *ocm*. A. Genomic region near 60C showing predicted genes and five BACs used to rescue *ocm*. The BACs shown in black failed to rescue *ocm* mutations, but 117N13 (yellow) rescued viability and fertility of multiple *ocm* alleles. B. Conceptual translation of *ocm* reveals a protein with distinct motifs conserved between other Drosophila species (colored boxes) separated by diverged regions (thin line). The hatched box indicates the segment of OCM used to raise antibodies. C. Similar coding regions are found in the genomes of some other insects. The Bombyx alignment is taken from genomic DNA lacking cDNA support, so the exact alignment is uncertain. D. The Cys431 region (red) is also found in chordate Mga. E. Several Daphnia proteins contain Cys431 motifs. A second cys-rich region (orange) is also present in two of the Daphnia proteins.

We mapped *ocm* mutations to polytene band 60C near the 2R telomere (Fig. 3A). The mutation fell inside Df(3R)BSC155, but outside Df(3R)BSC780 defining a region of roughly 120 kb containing about 25 genes. Df(3R)BSC155 also produced solid red eyes in our eye reporter assay of dosage compensation demonstrating that the mechanism is haploinsufficiency (data not shown). We were able to exclude some genes by complementation tests. To identify which of the remaining candidates corresponded to *ocm*, we made transgenics carrying a set of 20 kb BACs spanning the relevant interval [36]. BAC CH322-117N13 rescued the viability and fertility of null mutations (Fig. 3A). Sequencing genomic DNA from the mutant flies showed that *CG3363* was the relevant gene (Table S1A). *CG3363* was recently identified in a cell culture based RNAi screen as a strong repressor of MSL activity [30]. Most of the strong alleles (no adults recovered) were stop codons and most hypomorphs (adult daughters, but few or no sons) were missense mutations altering highly conserved codons (Fig. S2). A few mutations initially scored as hypomorphs were found to carry stop codons (Table S1B), but these conformed to the rules previously found to allow some translational readthrough [37]. We suspect low levels of full-length protein are made which could account for the traces of activity detected phenotypically.

Conceptual translation of the DNA sequence predicts a 2175 amino acid protein with no similar family members in *Drosophila* (Fig. 3B and C). A single homolog was found in the genomes of other *Drosophila* and some insect species. No similar sequences could be identified in the genomes of social insects (bees, ants, and wasps) that use a haploid-diploid strategy of sex determination. This failure is unlikely due to poor sequence assemblies because homologs for all the *msl* genes were easily found in these same species. Alignments between OCM containing insect species revealed a number of conserved motifs separated by highly diverged regions (Fig. 3B). BLAST analysis of the individual conserved domains failed to identify any matches outside the OCM homologs showing that it is not part of a larger gene family. No strong inferences regarding biological activity could be deduced from the conserved motifs. No vertebrate matches were found when using the full OCM sequence. We noticed a short tandem duplication of a cysteine rich motif that we named Cys431because the four cysteine residues were separated by four, three, and one amino acid residues (Fig. S3). Searches using only the Cys431 sequence identified a single vertebrate protein, MGA, a DNA binding protein that heterodimerizes with the MAX family of transcription factors [38]. Although an *MGA* gene can be found in all chordate genomes, only three regions are strongly conserved, an N-terminal T-box DNA binding motif, the Cys431 element in the middle, and a bHLH region responsible for MAX binding (Fig. 3D). The *Drosophila* OCM lacks any similarity to MGA outside the Cys431 sequence. Four genes from Daphnia contained Cys431. One of these, 222106, shares a second Cys-rich motif with OCM and has an N-terminal THAP DNA binding motif (Fig. 3E). In most genes the Cys431 motif is a tandem duplication of ∼15 amino acid residues, but in both Danaus (butterfly) and Bombyx (moth), a single unit is present (Fig. S3). Sharing paired Cys431 motifs with MGA and the Daphnia THAP domain protein might indicate that OCM is involved in some aspect of transcription regulation.

### OCM Distribution

**Figure 4:**
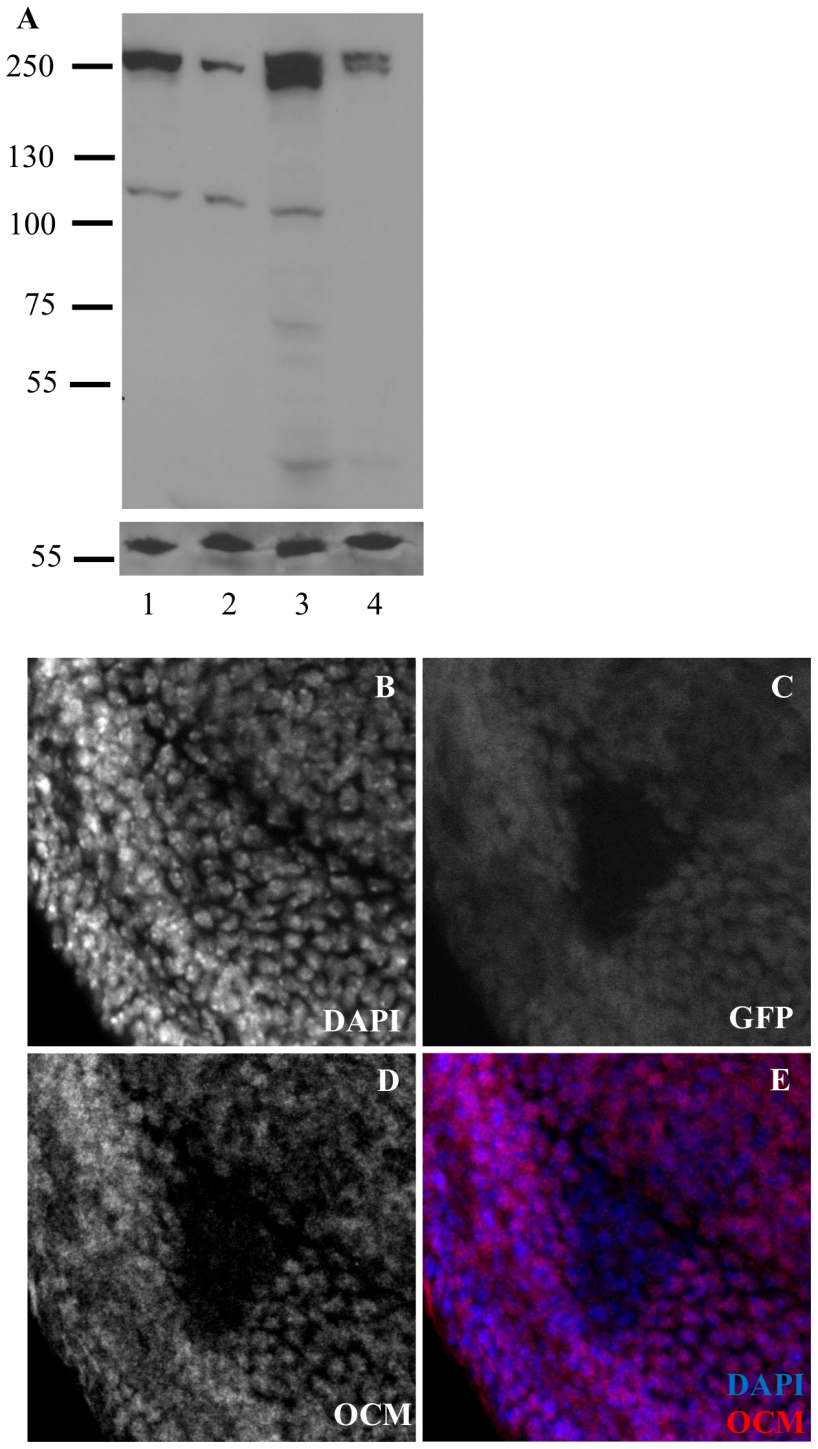
Antibodies to OCM recognize a 250 kD nuclear protein. A. Anti-OCM western with size standard shown left. Lane 1, wild type embryos. Lane 2, embryos from *Df ocm*/+ mothers. Lane 3, wild type adults. Lane 4, *Df ocm*/+ adults. Hemizygous tissue gives a weaker OCM band. Loading control (LC) is mitochondrial complex V. B-E. Third instar imaginal eye disc showing an *ocm*/*ocm* clone surrounded by *ocm*/+ cells.

Previous high throughput surveys showed that *ocm* transcripts are of low abundance [39], and OCM peptides were only recovered in P53 pulldowns along with many other partners [40]. In an effort to visualize OCM protein, we raised polyclonal antibodies against a segment of OCM. Codons 1287–1773 were overexpressed as a bacterial fusion protein to immunize guinea pigs (Fig. 3B). Affinity purified antibodies recognized a ∼250 kD band from embryonic and adult protein extracts (Fig. 4A). Although the molecular weight is similar to that predicted for OCM, we lack null tissue to show specificity. Instead we prepared protein extracts from hemizygous adults and embryos of hemizygous mothers (Fig. 4A lanes 2 and 4). In each case the 250 KD band became weaker despite similar amounts of protein loaded in each lane. In order to further validate the antisera, we stained imaginal discs carrying homozygous clones of the *ocm^P1048Z^* frameshift allele that should truncate OCM before the epitopes recognized by the antibody. Figures 4B–E show that OCM staining is detectable in nuclei of surrounding *ocm*/+ heterozygous cells, but OCM staining is greatly reduced within *ocm* null clones (Fig. 4D). These results show that the antiserum specifically recognizes OCM protein and that *ocm/ocm* cells continue to proliferate for several divisions as any old OCM protein is diluted by growth. The staining filled the nuclei of diploid imaginal disc cells in both sexes. No evidence for a more restricted localization over the X chromosome was seen although the small size of the cells limits the analysis. Staining wild type polytene chromosomes (squashed or unsquashed) from salivary glands with anti-OCM antibodies did not detect any reproducible signal in either sex (data not shown).

### Lethal Phase of *ocm* Null Mutations

Besides its role in dosage compensation, *ocm* has additional phenotypes that might provide clues to its biochemical function. Because we could detect low activity in a few of our mutants with early stop codons, we selected the original *ocm^P1048Z^* allele for lethal analysis because it is the only frameshift allele recovered (11 base pair deletion). Crossing *ocm^P1048Z^* to *Df(3R) BSC155* produced hemizygous progeny that did not develop past second instar larvae. Survival to this stage might depend upon maternally provided OCM, but we were unable to make *ocm* null eggs because the gene is needed for oogenesis (see below). Mutant larvae were lethargic but continued to move for several days before dying with grossly normal morphology.

**Figure 5:**
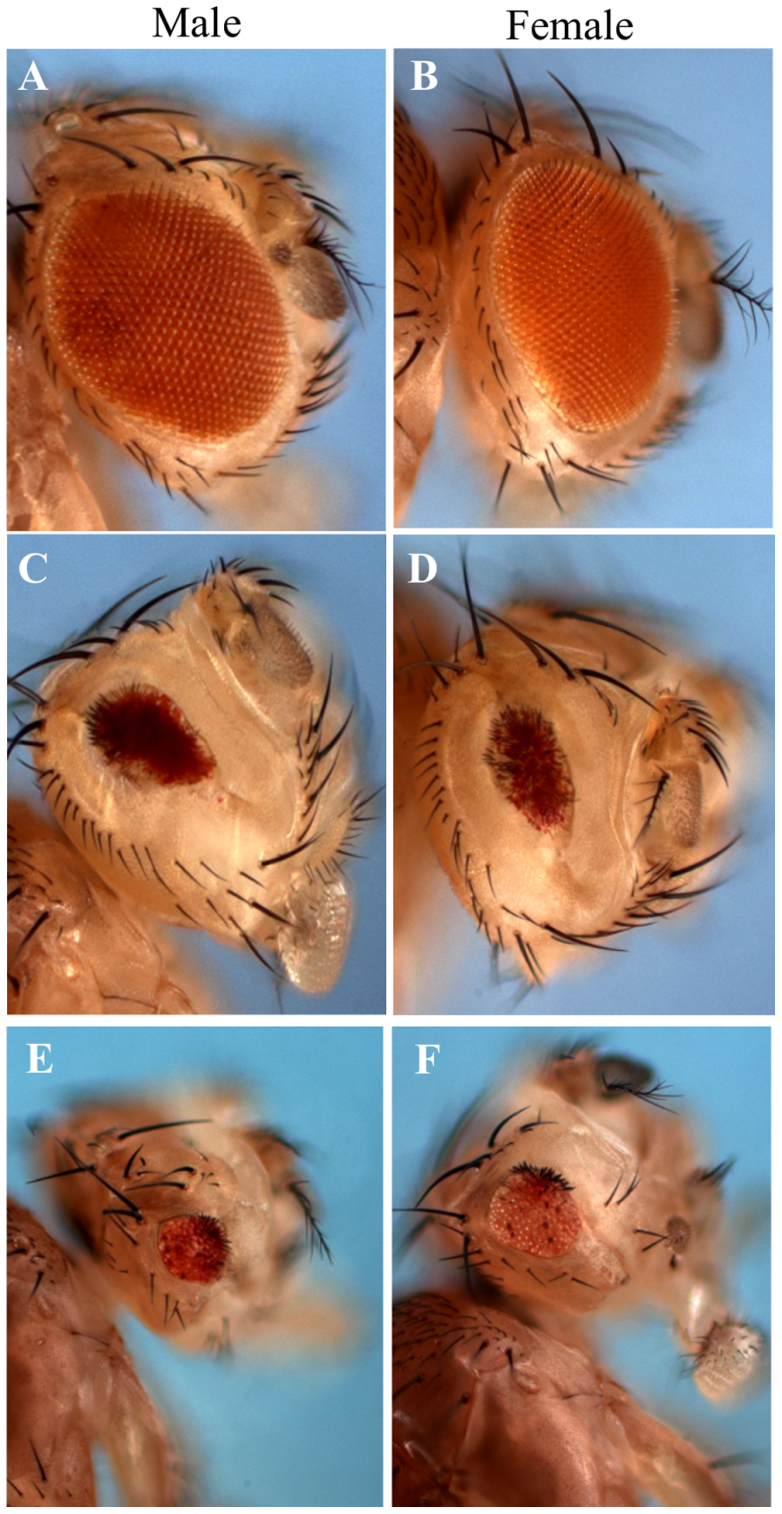
Null alleles of *ocm* results in decreased cell numbers in the fly eyes. Different *ocm* alleles were assayed for cell viability in *Drosophila* eye. (A-B) *ocm^S1590F^*, a weak hypomorphic allele of *ocm* does not affect cell growth. Other hypomorphic alleles, *ocm^G1646E^* and *ocm^V1286D^* were also tested but they are indistinguishable to *ocm^S1590F^* (data not shown). Increasingly severe alleles (C–D) *ocm^V1334D^*, (E–F) *ocm^166^* ^Δ*11 bp*^ (null allele) result in fly eyes dramatically reduced in size.

Although *ocm* null animals die at second instar, *ocm* null cells are able to form clones in imaginal discs (Fig. 4D). We asked whether such clones could go on to differentiate into adult tissues. We generated heterozygous *ocm*/+ animals whose eyes were composed almost entirely of *ocm*/*ocm* mutant clones (Fig. 5). The alleles chosen for analysis are homozygous lethal to both sexes, but female viable in combination with the very weak L1658N allele (Table S2). Each chromosome is free of secondary mutations as determined by the ability of BAC117N13 to fully rescue viability and fertility (data not shown). Adult eye morphology varied dramatically depending upon the *ocm* allele tested. Eye differentiation was normal with weak hypomorphs (Fig. 5A–B). However, the eyes became increasingly smaller as more severe *ocm* alleles were tested (Fig. 5C–F) although a few omatidia form even in *ocm* null mutants. Either *ocm* is not entirely essential for cell prolifertion or the wild type protein persists for several cell cycles after mitotic recombination has removed the wild type *ocm* allele. The presence of recognizable omatidia demonstrate that surviving mutant cells are able to differentiate without OCM. There was no difference in the clone size or eye morphology between the sexes (Fig. 5A–F). The shared morphology indicates that reduced tissue survival in this case is due to the loss of essential functions common to males and females, not excess dosage compensation.

### *ocm* Blocks Oogenesis

Adult females produced by hypomorphic *ocm* combinations were invariably sterile and failed to lay any eggs. Upon dissection the tiny paired ovaries were recognizable by their attachment to a common oviduct (Fig. S4A & B), but each whole ovary was smaller than a single wild type stage 10 egg chamber. When fixed mutant ovaries were stained with Alexa Fluor 488 phalloidin, the layer of muscles surrounding individual ovariole tubes was clearly visible, but no egg chambers were found inside (Fig. S4A and B). We tried to stain wild type egg chambers with anti-OCM antibodies but we could not detect any discernible staining (Fig. S4C). Our antibody also could not detect OCM from *ocm* ^L1658N^ homozygous females even though the missense mutation should produce a full-length polypeptide (Fig. S4B).

A few escaper adult males were recovered with our weakest allelic combinations, and they were also sterile. However, their testes appeared morphological normal and were filled with sperm (data not shown).

## Discussion

The MSL complex has been intensively studied as a model to understand how an RNA-dependent chromatin remodeling machine can produce a subtle, but essential two-fold change in global transcription across the male X chromosome. The MSL complex must act on hundreds of unrelated genes and successfully adjust their expression within a very narrow range. XXX:AA females die (50% too much X products) and XY:AA males mutant for any *msl* gene die (50% too little X products). We used a genetic strategy to search for new genes that might regulate the dosage compensation pathway to achieve this precise set point. The *miniwhite* eye color marker adjacent to *roX1* provided an easily scored phenotypic reporter for changes in MSL complex activity that are too small to affect male viability. Rather than look for subtle changes in the color of individual pigment cells, our assay relies on large changes in the fraction of eye tissue where the MSL complex successfully overcomes silencing of *miniwhite*.

We recovered 30 independent EMS alleles of the *ocm* gene (*CG3363*) that all produced similar increases in eye pigmentation from the MSL-dependent reporter exclusively in males. Most mutants were strong loss of function or complete nulls such that homozygotes of both sexes died by second instar larvae. However, the few hypomorphic missense alleles recovered were especially informative because they revealed a second dosage compensation phenotype, recessive male-specific lethality. Male lethality could potentially occur at any number of points in the dosage compensation pathway from the initial sex determination decisions, through production of MSL proteins subunits or *roX* RNAs, assembly of complex, targeting to active X-linked genes, or biochemical activity of the complex. For instance, ectopic MSL2 expression kills most females and renders the few escapers sterile, similar to *ocm* [41]. However, the observation that *ocm* females do not ectopically express MSL complex and *msl* mutations do not rescue *ocm* female toxicity disprove this idea. Excluding subtle defects in subunit production is more difficult, but several observations argue against this idea. First, separately reducing production of MSL1, MSL2, MLE or *roX1* RNA each partially rescues *ocm* males arguing that it is the full complex rather than one individual component that is affected. Similarly, *ocm* mutations do not affect *cis* spreading behavior of MSL complex from ectopic autosomal sites of *roX1* transcription. Spreading behavior is highly sensitive to the ratio of MSL proteins subunits to nascent *roX1* RNA suggesting individual components are produced in normal amounts and efficiently assembled into complexes [35], [42]. Examination of hypomorphic mutations that allow males to survive to third instar, but not reach adulthood, showed no gross defect in the finely banded pattern of MSL distribution along the X chromosome. We think it is unlikely that MSL localization is affected. One interpretation of these experiments is that MSL complex is produced and delivered to X-linked genes normally, but is overly active when *ocm* activity is reduced. One way this might occur is if OCM removed chromatin marks deposited by the MSL complex. However, the predicted OCM sequence shares no obvious similarity to known histone deacetylases, phosphatases, or deubiquitylases.

We asked if OCM protein might colocalize with MSL complex along the male X, but our antibodies were unable to detect it on both squashed and whole mount polytene chromosomes. No discernible staining in embryos (data not shown), S2 cells (data not shown) and developing egg chambers was observed either. One potential difficulty in working with OCM is its apparently poor expression levels across all developmental stages [39]. This makes differentiating authentic staining from background difficult, if not impossible. Only in mosaic tissues where *ocm* and wildtype cells are juxtaposed, any miniscule differences in staining can be attributed confidently to the presence of OCM (Fig. 4). For this reason, we conclude that *ocm* is predominantly a nuclear protein, at least in the imaginal discs. Nuclear localization is compatible with the presence of a Cys431 motif found in two unrelated DNA binding proteins, vertebrate MGA and Daphnia 222106. However, no obvious sex difference or localized concentration that might correspond to the X was seen.

OCM plays multiple roles in development judging from the pleiotropic phenotypes seen in the different alleles. The defect in spermatogenesis is most likely late because motile sperm are present in escaper *ocm* males, although we could only recover adult males from the weakest *ocm* alleles. By contrast, the defect in oogenesis is so early that no recognizable egg chambers are formed. Null mutants complete embryonic development but die as young second instar larvae. These female phenotypes clearly demonstrate that OCM is also required for pathways unrelated to the MSL complex. We looked to see if the modular pattern of amino acid sequence conservation might correspond to functionally distinct regions of the protein by determining the codons changed in many alleles. We did notice that the five missense alleles that allow female development but cause excessive MSL activity all cluster in two small, highly conserved segments. While these must be necessary for proper dosage compensation, they cannot be exclusively dedicated to that function because adult females also suffer early blocks to oogenesis. Several of the MSL subunits have recently acquired novel domains that are necessary for the specialized function of dosage compensation of the male X [14], [27]. We examined the pattern of amino acid sequence conservation between *Drosophila* species that use the MSL complex for dosage compensation of the male X compared to more distantly related insects that use the MSL proteins for other purposes. No obvious new adaptation was found in fly OCM. One explanation for the wider conservation of OCM motifs implicated in *Drosophila* MSL activity might be that OCM also modulates MSL activity in insects that use it for other pathways.

A recent RNAi screen for regulators of *roX2* expression in cultured cells identified *CG3363* as the strongest repressor of MSL activity [30]. At that time no phenotypic information was available for fly mutants, but our results show a specific genetic interaction with MSL complex in whole flies. The fact that *CG3363* appeared in two independent genetic screens using different strategies provides confidence that it is a newly recognized negative regulator of the dosage compensation pathway.

## Materials and Methods

### *Drosophila* Stocks and Fly Genetics

Larvae and flies were raised on standard cornmeal-yeast-agar-molasses medium containing propanoic acid at 25°C. In all experiments the *roX1* mutation is *roX1^ex6^* and the *roX2* allele is *Df (1) roX2^52^* [24]. The *[w^+^4*Δ*4.3]* transgene supplies essential adjacent genes lost in the *roX2* deletion.

### Genotypes

The full genotypes of the flies in Figure 1 are as follows:

A–B) *y w; [GmroX1-75C].*

C–D) *y w; ocm^L714X^/+*; *[GmroX1-75C].*

E–F) *y w;msl1+/+ ocm^L714X^; [GmroX1-75C].*

G–H) *y w; [GmroX1-58D].*

I–J) *y w; ocm^Q1673X^/+*; *[GmroX1-58C].*

K–L) *y w^m4^*.

M–N) *y w^m4^; ocm^G1646E^/+.*

O) The wildtype flies are *y w*. The *ocm* allele used for viability assay is indicated beside the graph.

### Mutagenesis

The mutagenesis was carried out as described in [14] except that all flies were homozygous for the [*w* ^+^ *GMroX1*] reporter at all times because pilot experiments showed the modifiers produced a more conspicuous phenotype when the reporter was paired. The *[GMroX1-75C]* and [*GmroX1-58D] t*ransgenic stocks used were also as previously described [14].

### Complementation Test

*CyO, y* ^+^ balanced males from each of the modifier mutants were mated to comparable females and the presence of nonbalanced progeny (yellow body and flat wings) was scored.

### Crosses

The crosses used for **2A.** *y w*; *ocm* ^G1646E^/*CyO*, *y* ^+^ virgins crossed to *y w*/Y; *msl ocm* ^L1658N^/*CyO*, *y* ^+^ males where *msl* is either *msl1* ^L60^, *msl2* ^1^, or *mle^1^*. The interesting class of progeny had flat wings and yellow body.

**2B.** The control classes were produced by *w*; *msl1* ^hypo^/*CyO, Roi* sib crosses to produce homozygotes or crosses to *y w*; *msl1* ^L60^/CyO to produce hemizygotes. The double mutants were made by mating *y w/Y*; *msl1* ^hypo^ *ocm* ^L1658N^/*CyO*, *y* ^+^ males to *y w*; *msl1* ^L60^ *ocm* ^L1658N^/*CyO*, *y* ^+^ virgins.

**2C.** *y w roX1 roX2* [w^+^ cos4Δ]; [*w* ^+^ *GMroX1-75C*] virgins crossed to *y w*/Y; *msl1* ^L60^ *ocm*/*CyO*, *y* ^+^ males where the different *ocm* alleles are showed for each column.

### Rescue Experiment

Standard P element transformation was used for BAC rescue experiments [43]. Five 20 kb BAC (bacterial artificial chromosomes), 113N09, 12L17, 08I22, 99L08, 117N13 derived from a P[acman] library made available from the Bellen lab, were used for the rescue [36]. Irrelevant secondary mutations were removed from *ocm* chromosomes by back crossing to the starting chromosome until BAC 117N13 could rescue *ocm* homozygotes to Mendelian frequencies.

### Sequencing *ocm* Mutations

The following primer pairs were used to amplify genomic DNA from adult flies followed by Sanger sequencing separately from both strands. Several background polymorphisms relative to the reference sequence were found during sequencing, but not shown. Likewise, a few nonsense mutations carried nearby missense mutations as well, but only the nonsense mutation is shown.

### Mitotic Clones

Six *ocm* alleles (*ocm^S1590F^, ocm^G1646E^ ocm^V1334D^*, *ocm^V1286D^*, *ocm^Q1297X^* and *ocm^166^* ^Δ*11 bp*^)were first recombined to the *[FRT42D]* transgene as described [44] Mitotic clones were then induced in the eyes by crossing them to *y w; [FRT42D] [y^+^]++ ocm/[FRT42D] [y^+^] [GMR-hid] [w^+^ cl]+*; *[ey-GAL][UAS-FLP]* as described [43] to assay for cell viability. For immunostaining, *y w*; *[FRT42D] ocm^Q1297X^* was crossed to *y w [ey-flp]; [FRT42D], [Ubi-GFP]/Cyo* or *y w [Ubx-flp]; [FRT42D], [Ubi-GFP]/Cyo* flies to generate mitotic clones in the eyes and wings discs respectively. The flies stocks were gifts from the Bellen’s lab.

### Immunostaining

Immunostaining on imaginal discs and embryos were performed as described [45]. The ovaries were mounted with Prolong gold (P-36931, Invitrogen) with no pre-treatment.

### OCM Polyclonal Antibodies

A segment of the gene spanning codon 1287–1773 was subcloned into the pMAL-C2X vector (N8076, NEB). The fusion protein was expressed and run on a SDS page acrylamide gel. The protein was excised from the gel and injected into guinea pigs (Cocalico Biologicals, Reamstown, PA). The animals were sacrificed and the anti-serum collected after 91 days.

### Western Blots

Adult flies and embryos were first dounced in 50 mM Tris-HCl (pH 8.5), 300 mM NaCl and 1% NP-40 containing protease inhibitor (04693124001, Roche) as described in [15]. The samples were then boiled with SDS loading dye for 5 min and loaded onto an 8% SDS-polyacrylamide gel. Proteins were transferred in Tris-glycine buffer (pH 8.3) containing 25 mM Tris and 192 mM glycine and 20% methanol, for 1.75 h at 600 mA. Membranes were incubated with OCM antibodies overnight at 4°C. After three 15-min washes in PBS-Tween and 2 h incubation at room temperature with the appropriate secondary horseradish peroxidase conjugate, the proteins were detected with luminol system (Santa Cruz Biotechnology). The membrane was then stripped in 60 mM Tris-HCl pH 6.8, 0.7% β-mercaptoethanol and 2% SDS and reprobed in anti-ATP5A antibody (MS407, ABCAM).

## Supporting Information

Figure S1 ***Ocm*** **do not affect the morphology of the X chromosomes or spreading.** To check for gross morphology of the X chromosome, an attempt was made to retrieve third instar larvae from L1658N/S1590F (weakest combination), L1658N/G1646E, L1658N/W1401X and S1590F/Q1297X (strongest combination) crosses for MSL1 immunostaining. The morphology of the X in L1658N/W1401X larvae is normal in both A) males and B) females. We did not see altered X morphology in L1658N/S1590F and L1658N/G1646E as well (data not shown). No male larvae could be retrieved from S1590F/Q1297X and L1658N/W1401X is the most severe *ocm* heteroallelic combination where male third instar larvae can be recovered (See Table 1 for comparison). We also assayed the ability of *ocm* to affect spreading of the MSL complex around the *GmroX1* transgene inserted at 75C. C) The MSL complex binds to the *roX1* transgene and appears as a single sharp band (red arrow). D) Reducing 50% *ocm* activity does not affect the ability of the transgene to recruit MSL complex (red arrow). E) The MSL complex spreads megabases from the same *GmroX1* transgene when the fly is mutant for *roX1* and *roX2* (yellow line). F) Spreading seems to be unaffected after reducing 50% *ocm* activity (yellow line). The genotype for the larvae is C) *y w; [GmroX1-75C]/+,* D) *y w; ocm* ^L714X^ */+; [GmroX1-75C]/+,* E) *y w roX1 roX2; [GmroX1-75C]/+,* F) *y w roX1 roX2; ocm* ^L714X^ */+; [GmroX1-75C]/+*.
(DOCX)

Figure S2 **Hypomorphic mutations in** ***ocm*** **.** The region corresponding to the black box in Figure 3B and the location of three missense mutations in highly conserved codons (white letters on black background).
(DOCX)

Figure S3 **Sequence alignment of Cys431.** Upper half shows the sequences of insect OCM. The central two proteins from lepidoptera contain only one copy of the cys repeat. The lower half shows MGA proteins from chordates. The last four sequences are from Daphnia proteins. Daphnia 222106 has the best match to the consensus sequence, a second cys-rich region shared with OCM (not shown) and a THAP DNA binding motif.
(DOCX)

Figure S4 ***ocm*** **is needed for oogenesis.** All three samples are shown at the same magnification. A. Paired ovaries from L1658N mutant are bundles of empty ovarioles (stained with phalloidin). B. Same ovaries stained with anti-OCM. C. Individual dissected wild type ovariole containing a germarium through stage 8 egg chamber stained with anti-OCM. We could not detect any discernible OCM staining in any part of the tissues.
(DOCX)

Table S1 A. Thirty alleles of *ocm* were isolated in three separate EMS screens. The first three alleles were isolated with [*w* ^+^ *GMroX1-75C*] as were the next 16 alleles. The last eleven alleles were isolated using [*w* ^+^ *GMroX1-56D*]. The alleles that were sequenced are indicated with the first letter indicating the wild type codon, the codon number, and the replaced amino acid with X = stop codon. All of the missense alleles retained some activity as did several of the stop codons when placed in combination with missense alleles. B. Hypomorphic nonsense mutations. Female viability relative to the balancer sisters recovered in the same cross when mated to two weak hypomorphic alleles (805 and 127). Five of the six nonsense alleles have a cytosine base (underlined) followed by the stop codon. This allows low levels of translational readthrough producing low OCM activity making them hypomorphs. The bottom nonsense allele is followed by a guanine, produced no escapers and hence, classified as a null allele.
(DOCX)

Table S2 Complementation table of a subset of *ocm* alleles. *y w*; *ocm* ^a^/*CyO y* ^+^ mothers were crossed to *y w/Y*; *ocm* ^b^/*CyO y* ^+^ fathers. Any viable progeny were recognized by yellow body color and flat wings. For each cross the actual number of *CyO y* ^+^ sons (B) and daughters (D) are given along with the % viability of *ocm* ^a^/*ocm* ^b^ sons (A) and daughters (C) where the expected ratio is 1∶2, *ocm*: *CyO*. Most heteroallelic combinations were lethal to both sexes, but in some combinations (gray fill) daughters were viable but all sons died. In all cases the recovered females failed to produce any eggs. Recombination was used to remove secondary lethal mutations from chromosomes carrying missense mutations. Success was measured by the ability to recover homozygous *ocm* progeny in Medelian ratios that carried the 117N13 *ocm* ^+^ transgene. That allowed the viability of homozygotes lacking the transgene to be measured (dark boxes).
(DOCX)
