# Supplementary figures and images for: The Drosophila Over Compensating Males Gene Genetically Inhibits Dosage Compensation in Males
Source: PLoS One. 2013 Apr 2;8(4):e60450. doi: 10.1371/journal.pone.0060450 (PMC3615101; doi:10.1371/journal.pone.0060450)

**
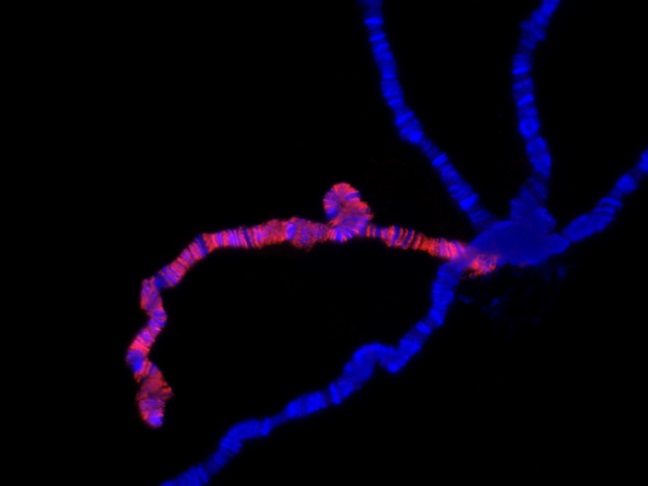

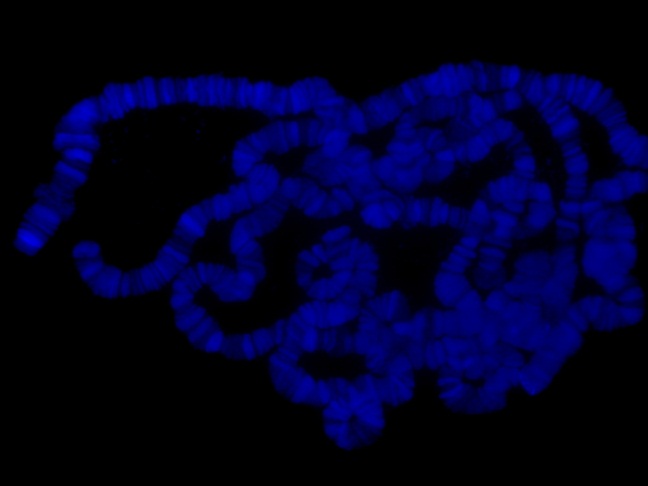
**

**B**

**A**


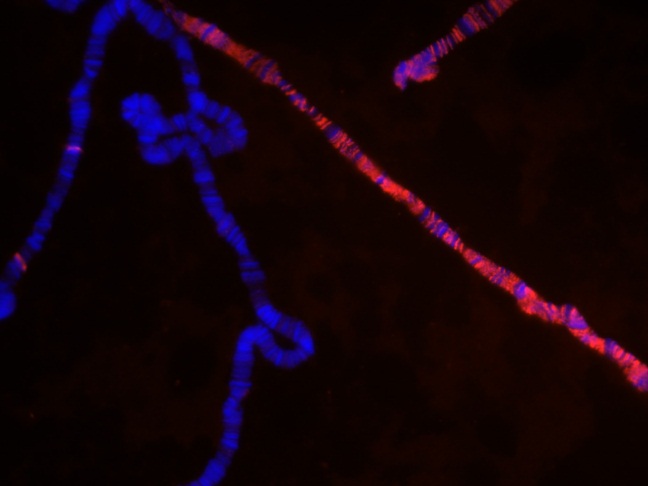


**D**


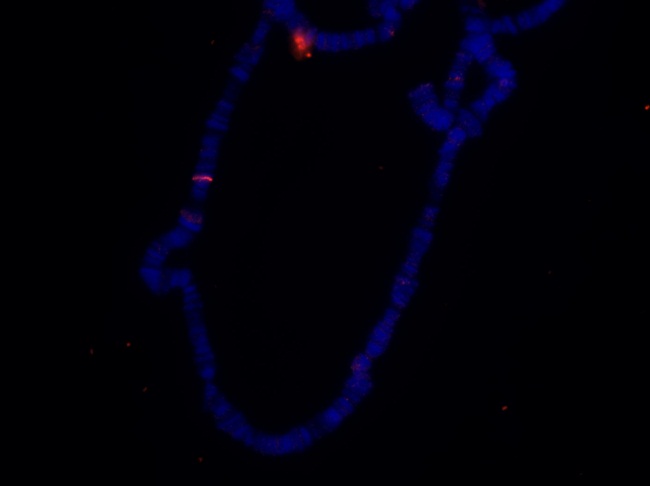


**C**


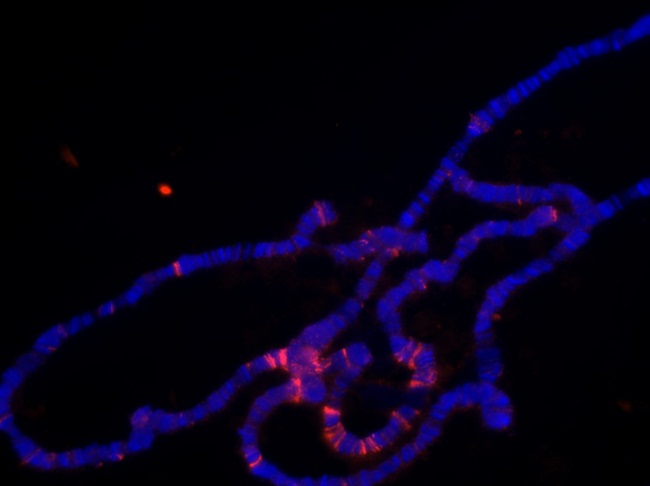


**F**


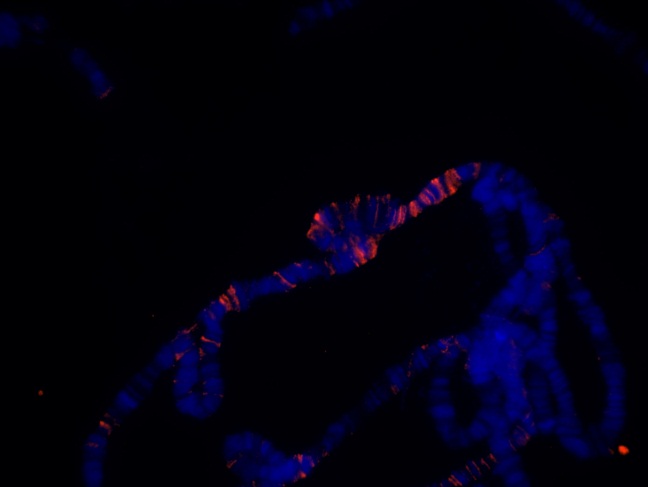


**E**

Supplement: Figure S1 — Ocm do not affect the morphology of the X chromosomes or spreading. To check for gross morphology of the X chromosome, an attempt was made to retrieve third instar larvae from L1658N/S1590F (weakest combination), L1658N/G1646E, L1658N/W1401X and S1590F/Q1297X (strongest combination) crosses for MSL1 immunostaining. The morphology of the X in L1658N/W1401X larvae is normal in both A) males and B) females. We did not see altered X morphology in L1658N/S1590F and L1658N/G1646E as well (data not shown). No male larvae could be retrieved from S1590F/Q1297X and L1658N/W1401X is the most severe ocm heteroallelic combination where male third instar larvae can be recovered (See Table 1 for comparison). We also assayed the ability of ocm to affect spreading of the MSL complex around the GmroX1 transgene inserted at 75C. C) The MSL complex binds to the roX1 transgene and appears as a single sharp band (red arrow). D) Reducing 50% ocm activity does not affect the ability of the transgene to recruit MSL complex (red arrow). E) The MSL complex spreads megabases from the same GmroX1 transgene when the fly is mutant for roX1 and roX2 (yellow line). F) Spreading seems to be unaffected after reducing 50% ocm activity (yellow line). The genotype for the larvae is C) y w; [GmroX1-75C]/+, D) y w; ocm L714X /+; [GmroX1-75C]/+, E) y w roX1 roX2; [GmroX1-75C]/+, F) y w roX1 roX2; ocm L714X /+; [GmroX1-75C]/+. (DOCX) [file pone.0060450.s001.docx]
