# Supplementary material for: The Drosophila Over Compensating Males Gene Genetically Inhibits Dosage Compensation in Males
Source: PLoS One. 2013 Apr 2;8(4):e60450. doi: 10.1371/journal.pone.0060450 (PMC3615101; doi:10.1371/journal.pone.0060450)
Supplement: Figure S2 — Hypomorphic mutations in ocm . The region corresponding to the black box in Figure 3B and the location of three missense mutations in highly conserved codons (white letters on black background). (DOCX) [file pone.0060450.s002.docx]

260A S1590F

Danaus LSKWFLMTIVNDFTEIRFYNKGFFVKYDSVIKAINVARISGKTVRLSSQRCVTAQS----

Bombyx CSKWFMMTVENDFSEIRFNNGGFFVTYDKIVKAINVARISGKAVRLSSQKCAPKNS----

tribolium FCRWRMIYLNSDFSYLNFTKSNYSIKYTDLLDVVKMASTVRSTITLRNQEIRQFYKTYTF 1639

Aedes MRRWFMLNVVNDFSDIYIPSWKSCLAYARIKQAIQLANRYNKTVRLTSIVQKNNQA---- 1818

Culex SRRWFMLTIVNDFSDIYIPSWKSCLTYGRIMQAIQLANRYRKTVKLTSIVLKNE------ 1739

Anoph HCRWIKLYIVDDFTDMYIPSWRYCIQHSQLMKAIQHANRHRAVRCLEFTLSPNNRSTKRS 2260

Mayetiola TQRLLRMHIGDDFTHMWFPTWRKFLSLKQIQNTLTMAKASGKAIEMSQSVNRPK------

moj DHRWLVLNLYDDFSHIFVPAFKDMISLDRIHNVARVAHDTNKMVKLQFFQDAPYD----- 1736

vir DHRWLVLNLYDDFSHIFVPAFKDMISLDRIHNVVRVAHDTEKIVKLQFFQDAPYD----- 1640

grim DHRWLVLNLYDDFSHIFVPAFRDMISLDRIHNVMHVAQDTRKVVKLQFFQDAPYD----- 1663

yak DHRWVVLDIFDDFSHIFVPSFSDMISLTRIHSVMQVAEERQKVVKLQFFPNAPYD----- 1634

erecta DHRWVVLDIFDDFSHIFVPSFSDMISLTRIHSVMQVAEERQKVVKLQFFPNAPYD----- 1642

mel DHRWVVLDIFDDFSHIFVPSFSDMISLTRIHSVMQVAEERQKVVKLQFFPNAPYD----- 1631

ana DHRWLVLDLFDDFSHIFVPALGDMISRDRIHSVMEVAVEKKKVVKLQFFQNAPYD----- 1604

persim DHRWVVLDLVDDFSHIFVPAFGEMISLDRIHKVMRVARDKNKLVKLQFFHQAPYD----- 1657

pseudo DHRWVVLDLVDDFSHIFVPAFGEMISLDRIHKVMRVARDKNKLVKLQFFHQAPYD----- 1659

will DHRWVVLDLYDDFSHIYVPAFRDMISLDRIHNVMRVSSEKQKVVKLQFFQNAPYD----- 1688

610A G1645E 127 L1658N 9 Q1673X

Danaus DPQFGIYAIPSKEKYCVFIGPYENN------ERLGIETVKNSV-VRKIKD-------------

Bombyx GPQYGLYAIPDSNEYCVFIGAYEPN------ESLGIQTIKTILHLRKQN------------- tribolium G----IFCVPDL-QDRIFVGPYLKTENHNVETLRYLQ---GKLIDTETFNKIIGKKFNE-- 1690

Aedes DVLPQIYAAPMQ-GECIFLGPYSYTQN---IDLILCQNVEGKMYTREEYERNNHIVRS---D 1873

Culex DVLPQIYAAPGQ-GDCIFLGPYSYTQT---IDLMLCQNVDGKMYTREEYERNNHIVRT---E 1794

Anoph RILPRIYAAPYQ-DNSIYVGPFPITQTQ--TDIMLCVASDGLLYTREEHERRHGIAVDSGAK 2319

Mayetiola ------VFVSHLHDNQVFMGPYESTDVQQNVSLFVR-NEERRMELAEDYHKKHGPKYTK

moj -----AFITPSS-KRVIYFGPLPLD--TPPPVLVLLQSVDGKMMLREVYQRNHNIPVQREK 1789

vir -----AFVTPSS-RRKIYFGPLRLD--MQPPVLVLLQSVDGKMMLREVYQRDHNIPVQRDR 1693

grim -----AFVTPLS-KRKIYFGPLRLD--MQPPVLVLLQSVDGKMMLREVYQRDHNIPVQHDR 1716

yak -----AFVTPSS-KKKIYFGPLSLD--MPPPVLVLLQSVDRKMMLREVYQREHSIPVQRHR 1687

erecta -----AFVTPSS-KKKIYFGPLSLD--MPPPVLVLLQSVDRKMMLREVYQREHSIPVQRHR 1695

mel -----AFVTPSS-KKKIYFGPLSLD--MPPPVLVLLQSVDRKMMLREVYQREHSIPVQRHR 1684

ana -----AFVTPSS-RKKIYFGPLHRNR-WPPPVLVLLQSVDGKVMLREVYQRAHSIPVDRQR 1658

persim -----AFVTAAS-RKKIYFGPLRLD--MSPPVLVLLQSVDGKMMLREVYQREHSIPVQPHH 1710

pseudo -----AFVTAAS-RKKIYFGPLRLD--MSPPVLVLLQSVDGKMMLREVYQREHSIPVQPHH 1712

will -----AFVTPSS-RRKIYFGPLKLD--MKPPVLVLLQSVDGKMMLREVYQKEHSIPVQRNR 1741
