# Supplementary material for: The Drosophila Over Compensating Males Gene Genetically Inhibits Dosage Compensation in Males
Source: PLoS One. 2013 Apr 2;8(4):e60450. doi: 10.1371/journal.pone.0060450 (PMC3615101; doi:10.1371/journal.pone.0060450)
Supplement: Figure S3 — Sequence alignment of Cys431. Upper half shows the sequences of insect OCM. The central two proteins from lepidoptera contain only one copy of the cys repeat. The lower half shows MGA proteins from chordates. The last four sequences are from Daphnia proteins. Daphnia 222106 has the best match to the consensus sequence, a second cys-rich region shared with OCM (not shown) and a THAP DNA binding motif. (DOCX) [file pone.0060450.s003.docx]

AEDS----KPRCDERYCGKGCICDVFKH---G-KVSRTHCSKLDCIFGCQCGYDKKRS Aedes

ASGDQGSRKRRCDERYCARGCICDVFRR---KPKGVAPHCNSIECVFGCSCGFDRKQQ Culex

VTDPSKSVHRPCDPLYCAKGCICDVLGW---SSESRTSHCQRIDCVLGCVCGYEQKQV Anopheles

SKSSEQCTKPFCRLGCVCESIEA----KKYIPSHCNRVECIFDCKCIDVSFK Mayetiola

AAARKADANKPCSKDYCHLGCICASLAG---TELPVRDHCGRAECVLDCRCLSAEESR D. melanogaster OCM

AAAIAKDANKPCSKDYCQLGCVCESVGG----DLAPREHCGRADCVLECSCTGAELTR mojavensis

AAAIAKDANKPCIKDHCQLGCLCESLAG----ELPAREHCGRADCVLACRCTGAELTR virilis

AAAIAKDANKPCIKDFCRLGCVCESLVG----VRPTREHCGRAECVLECRCTGTELTR grimshawi

AAARKADANKPCSKDHCQLGCLCASLAG---TELPVRDHCGRAECVLDCRCLSAEQGR yakuba

AAARKADANKPCSKDHCQLGCLCASLAG---TELPVRDHCGRAECVLDCRCLSAEQGR erecta

AAAARADEKKPCVKDYCQLGCVCDSLAG---TEIPLKDHCGRADCVLECCCIGGEHAR ananassae

AAARNADAKKPCIKEHCQLGCLCASLSG---AELPMRDHCGRADCVIECHCLGGEQSR persimillis

AAARNADAKKPCIKEHCQLGCLCASLSG---SELPMRDHCGRADCVIECHCLGGEQSR pseudoobscura

ADAKDADANKPCGKPYCQLGCVCASLAG---TDLPVRDHCAQAECVLNCRCLGGEQSR willistoni

--VATGTEDKPCSKSYCKMGCVCRSLLC---E--VFSDHCQMVDCMFGCKCPPSDTSS tribolium

TIPKTSEPSKSDKCFK-------------ACGRLECMFNCKCDFSKYNT Danaus

HCGLIECMFECKCDFSDYRT Bombyx

KRAPPCNNDFCRLGCVCSSLA----LEKRQPAHCRRPDCMFGCTCLKRKVVLVK human MGA

KRAPPCNNDFCRLGCVCSSLA----LEKRQPAHCRRPDCMFGCTCLKRKVVLVK cow

KRAPPCNNDFCRLGCVCSSLA----LEKRQPAHCRRPDCMFGCTCLKRKVVLVK mouse

RRAPPCNNDFCRLGCICASLA----LEKRQPTHCRRPDCMFGCTCLKRKVLVVK chicken

KRAPPCNNDFCRLGCVCASLA----LENRQPAHCRRPDCMFGCTCLKRKVMLVR platypus

KQISPCNKLFCRLGCVCQSLN----NKEHNFAHCRHADCMLECSCIQEKQLLEK xenopus

RRAPPCLNVFCRLGCVCASLV----HLRR-HHHCGKPQCMLGCSCLRRKVVALK Danio

QEKPPCNNNFCRLGCVCDSIK-DTGDASCEKEHCGKVDCMFGCVCTEEEKDESR lancelet

LPTVDCGQLFCRLGCICDTLVKTRNRPKMSMEHCGLPECMLQCVCGYQQGKPSS Daphnia106

RLTDECKKRFCTMGCICDSLN----STRPFYEHCNHPECMFYLVCSYKHNLRYR Daphnia661a

QSVVECGNRFCIMGCVCSSLH----LLKPRSQRCLHMECMFDHACIIKSKSTPS Daphnia661b

SNPCQTKKLCCELGCICSSLEN---AGKLPVEHCKNPDCMLEPSCTRSGDPSHT Daphnia359
