# Supplementary material for: The Drosophila Over Compensating Males Gene Genetically Inhibits Dosage Compensation in Males
Source: PLoS One. 2013 Apr 2;8(4):e60450. doi: 10.1371/journal.pone.0060450 (PMC3615101; doi:10.1371/journal.pone.0060450)
Supplement: Table S1 — A. Thirty alleles of ocm were isolated in three separate EMS screens. The first three alleles were isolated with [w + GMroX1-75C] as were the next 16 alleles. The last eleven alleles were isolated using [w + GMroX1-56D]. The alleles that were sequenced are indicated with the first letter indicating the wild type codon, the codon number, and the replaced amino acid with X = stop codon. All of the missense alleles retained some activity as did several of the stop codons when placed in combination with missense alleles. B. Hypomorphic nonsense mutations. Female viability relative to the balancer sisters recovered in the same cross when mated to two weak hypomorphic alleles (805 and 127). Five of the six nonsense alleles have a cytosine base (underlined) followed by the stop codon. This allows low levels of translational readthrough producing low OCM activity making them hypomorphs. The bottom nonsense allele is followed by a guanine, produced no escapers and hence, classified as a null allele. (DOCX) [file pone.0060450.s005.docx]

A.

| Pilot screen 75C | |  | Large 75C | |  | 56D screen | |  | |
| --- | --- | --- | --- | --- | --- | --- | --- | --- | --- |
| Isolation # | Sequence | | Isolation # | Sequence | Isolation # | | sequence | |  |
| 38 | K134X | | 122M |  | 9 | | Q1673X | |  |
| 127 | L1658N | | 142K | L825X | 31 | |  | |  |
| 166 | P1198Δ11 bp | | 231K |  | 56 | |  | |  |
|  |  | | 260K | S1590S | 178 | | V1334D | |  |
|  |  | | 310A |  | 273 | | V1286D | |  |
|  |  | | 430L | W1401X | 310 | | Q678X | |  |
|  |  | | 432L | Q1297X | 384 | |  | |  |
|  |  | | 451K | K1034X | 396 | | K1363X | |  |
|  |  | | 520G |  | 407 | | L714X | |  |
|  |  | | 540C |  | 482 | |  | |  |
|  |  | | 550A |  | 765 | |  | |  |
|  |  | | 610A | G1646E |  | |  | |  |
|  |  | | 623E | Y636X |  | |  | |  |
|  |  | | 760A |  |  | |  | |  |
|  |  | | 781A |  |  | |  | |  |
|  |  | | 805A |  |  | |  | |  |
|  |  | | 810B | Y888X |  | |  | |  |

B.

| Allele | Viability with (%) | Viability with (%) |
| --- | --- | --- |
| 38 | 805A | 127 |
| ATG AAG CAG  ATG **T**AG CAG | 24 | 75 |
|  |  |  |
| 623A |  |  |
| CCC TAT CTC  CCC TA**A** CTC | 43 | 0 |
|  |  |  |
| 310 |  |  |
| CCT CAG CCG  CCT **T**AG CCG | 0.3 | 0.4 |
|  |  |  |
| 432L |  |  |
| CTT CAG CGT  CTT **T**AG CGT | 37 | 1 |
|  |  |  |
| 430L |  |  |
| TTC TGG CTA  TTC T**A**G CTA | 1 | 2 |
|  |  |  |
| 9 |  |  |
| TAC CAG CGC  TAC **T**AG CGC | 24 | 55 |
|  |  |  |
| 810A |  |  |
| CGC TAT GAA  CGC TA**A** GAA | 0 | 0 |
