# Supplementary material for: The Drosophila Over Compensating Males Gene Genetically Inhibits Dosage Compensation in Males
Source: PLoS One. 2013 Apr 2;8(4):e60450. doi: 10.1371/journal.pone.0060450 (PMC3615101; doi:10.1371/journal.pone.0060450)
Supplement: Supplementary file 5 [file pone.0060450.s006.docx]

	Df 155		805		Q1673X		L1658N		G1646E		S1590F		W1401X		V1334D		Q1297X		V1286D		K134X	
K134X	0	192	0	461	0	163	0	377	0	182	0	570										
	0	226	24	630	1	297	76	433	0	220	0	638										
L714X			0	51			0	28	0	215	0	73					0	98				
			0	68			0	60	0	238	0	92					0	107				
P1198Δ11			0	389																		
			43	541																		
V1286D	0	149	131	153			85	523	3	473	21	442			0	557			0	241		
	0	167	125	237			104	617	29	480	57	437			0	763			0	303		
Q1297X	0	160	0	184			0	331	0	76	0	74										
	0	196	37	231			1	321	0	143	0	65										
V1334D			0	233			0	1182	0	726	0	635			0	131						
			28	303			0	1239	0	747	0	657			0	202						
K1363X	0	598	0	184																		
	0	754	27	247																		
W1401X	0	409	0	223	0	747	0	506	0	579	0	486									% exp N	*CyO*
	0	388	0.7	279	0	674	2	483	0	640	0	449										
S1590F	0	264	34	451	0	570	2	796			0	71								sons	A	B
	0	288	89	496	0	638	58	908			0	80								daughters	C	D
G1646E	0	311	8	494	0	177	0	245	0	553												
	0.5	426	68	616	0	200	52	292	0	475												
L1658N	0	260	61	124	0	774	38	581														
	0	268	100	128	55	1309	96	695														
Q1673X			0	321																		
			28	501																		
122M			0	603	0	124	0	1050	0	177	0	201	0	747	0	535	0	473	0	50		
			39	627	0	164	12	1083	0	200	0	211	0	674	0	602	0	518	0	84		
231K			0	220	0	197	0	159	0	380	0	162					0	200			0	204
			55	225	0	212	1	168	0	374	0	176					0	206			0	232
310A			0	138					0	182	0	217					0	213				
			1	146					0	198	0	281					0	248				
760A			0	261					0	646	0	270					0	118				
			0	298					0	729	0	305					0	133				
781A	0	394	0	49	0	492	0	567	0	487	0	365	0	315			0	637			0	345
	0	472	0	95	0	491	1	651	0	499	0	389	0	334			0	648			0	387
805A			19	253																		
			68	304																		
